# A Natural Approach to the Prevention and Treatment of Gingivitis and Periodontitis: A Review of Pomegranate’s Bioactive Properties

**DOI:** 10.3390/life14101298

**Published:** 2024-10-13

**Authors:** Georgiana Ioana Potra Cicalău, Laura Grațiela Vicaș, Gabriela Ciavoi, Timea Claudia Ghitea, Nagy Csaba, Roxana Alexandra Cristea, Florina Miere (Groza), Mariana Ganea

**Affiliations:** 1 Department of Dental Medicine, Faculty of Medicine and Pharmacy, University of Oradea, 1st Decembrie Street, 410073 Oradea, Romania; cicalau.georgiana@uoradea.ro; 2 Department of Pharmacy, Faculty of Medicine and Pharmacy, University of Oradea, 1st Decembrie Street, 410073 Oradea, Romania; lvicas@uoradea.ro (L.G.V.); mganea@uoradea.ro (M.G.); 3 Doctoral School of Biomedical Science, University of Oradea, No. 1 University Street, 410087 Oradea, Romania; nagycsaba95@yahoo.com (N.C.); roxicristea98@yahoo.com (R.A.C.); 4 Department of Preclinics, Faculty of Medicine and Pharmacy, University of Oradea, 410068 Oradea, Romania; florinamiere@uoradea.ro

**Keywords:** *Punica granatum*, pomegranate, gingivitis, periodontitis, oral health, antibacterial, antioxidant, inflammation

## Abstract

(1) Background: This systematic review explores the bioactive properties of *Punica granatum* (pomegranate) and its potential applications in the prevention and treatment of gingivitis, periodontitis, and other oral diseases. (2) Methods: A comprehensive literature search was conducted using PubMed and Google Scholar, focusing on pomegranate and oral diseases. Inclusion criteria included studies evaluating the effects of pomegranate on oral health, while exclusion criteria eliminated non-peer-reviewed and non-English articles. This review aims to assess the efficacy of pomegranate extracts as a natural alternative to synthetic pharmaceuticals in oral health care. A structured search strategy included key terms such as “pomegranate”, “oral health”, “gingivitis”, and “periodontitis”. A total of 125 relevant references were reviewed to identify the most pertinent findings. (3) Results: The results indicate that pomegranate extracts have demonstrated efficacy in reducing plaque, inhibiting harmful oral microorganisms, and promoting overall oral health. Furthermore, clinical studies highlight the potential of pomegranate-based products, such as mouthwashes and gels, as viable alternatives to conventional pharmaceuticals, particularly in resource-limited settings. However, the review also notes the need for further research, particularly in the form of clinical trials, to establish optimal formulations and long-term safety. (4) Conclusions: Pomegranate presents a promising, natural solution for preventing and treating gingivitis and periodontitis. Further studies should focus on long-term effects and clinical efficacy.

## 1. Introduction

Nutraceuticals, as defined by Dr. Stephen DeFelice, are food products that provide health benefits, including disease prevention or treatment. With growing concerns over antimicrobial resistance and side effects associated with synthetic treatments, natural alternatives such as pomegranate (*Punica granatum*) are becoming increasingly attractive for oral health care. Pomegranate has been traditionally used in Middle Eastern and Indian medicine, offering antibacterial, anti-inflammatory, and antioxidant properties. This review aims to evaluate the potential of pomegranate extracts in treating gingivitis and periodontitis [1,2]. These products encompass a broad range of categories, including herbal products, dietary supplements, processed foods, isolated nutrients, specific diets, and genetically modified “designer” foods [3,4].

Nutraceuticals have been studied extensively across various medical fields, with increasing interest in their application for dental and oral health issues [5,6]. They consist of biologically active molecules derived from food, which function similarly to both nutrients and pharmaceuticals [7,8]. Due to their diverse bioactive components, nutraceuticals offer significant medicinal value with minimal side effects [9]. In contrast, the overuse and misuse of synthetic microbial agents and antibiotics have led to antimicrobial resistance and the emergence of infections previously considered rare. Natural phytochemicals have demonstrated promise as effective alternatives to synthetic agents [10].

Several natural plant extracts, such as *Curcuma zedoaria*, *Calendula officinalis, Aloe vera, Origanum vulgare*, and *Magnolia officinalis*, have been shown to be effective in the prevention or treatment of oral diseases [11,12,13]. The ethno-medical history of pomegranates (*Punica granatum*) positions them as one of the most researched medicinal plants, with growing interest in their use in dental phytotherapy [14].

*Punica granatum*, commonly known as pomegranate, belongs to the Punicaceae family [15]. The genus name, Punica, originates from the Roman name for Carthage, while the name “pomegranate” is derived from the Latin words “pomum” (apple) and “granatus” (seeded) [16]. A specific subspecies, *Punica granatum var. pleniflora* (also known as golnaar), is notable for being the male version of the plant, which produces flowers but no fruit [17].

Some of the most well-known pomegranate varieties include Wonderful from the United States, Hicanzar from Turkey, Acco from Israel, Bagua from India, Valenciana and Mollar del Elche from Spain, as well as a dwarf variety, *Punica granatum* Nana, often used as an ornamental plant [18].

Pomegranates come from a large shrub, reaching heights of 3–5 m, with many spiny branches and glossy, lance-shaped leaves. As the tree matures, its bark turns gray. The flowers are large and can be red, white, or variegated, with a tubular calyx that eventually develops into fruit. The ripe fruit is roughly five centimeters in diameter, with a deep red skin and a pointed calyx. It contains numerous seeds, each surrounded by tart, red juice and separated by a white, membranous pericarp [16].

Native to Asia, various parts of the pomegranate shrub have been used traditionally for their anti-inflammatory, astringent, and hemostatic properties [15]. In the traditional medicine of the Middle East and India, pomegranate has been used for centuries as a medicinal fruit, a source of nutrition, and in cosmetics [19]. The tree was first domesticated in ancient Mediterranean regions characterized by mild winters and hot, dry summers, which are ideal for its growth [20]. Known as the “jewel of winter”, the pomegranate has been lauded for its disease-fighting abilities, largely attributed to its potent antioxidant properties [19]. It has a significant history in Persian medicine and is frequently referenced in historical medical and pharmaceutical literature [17].

The goal of this review is to assess the efficacy of *Punica granatum* as a natural alternative to synthetic pharmaceuticals in oral health care. Hypothesis: Pomegranate-based products will demonstrate significant antibacterial and anti-inflammatory effects on oral diseases such as gingivitis and periodontitis. Null hypothesis: Pomegranate extracts will not show a significant difference in treating oral diseases compared to synthetic treatments.

## 2. Materials and Methods

Review Type: Systematic review based on a structured search strategy.

Search Strategy: A literature search was conducted in PubMed and Google Scholar using combinations of terms like “pomegranate”, “*Punica granatum*”, “oral diseases”, “gingivitis”, “periodontitis”, and “antibacterial effects”. Inclusion criteria were peer-reviewed studies focused on pomegranate and oral diseases, published in English, and involving in vitro, in vivo, or clinical trials. Exclusion criteria (Table 1) removed non-peer-reviewed studies, articles not related to oral health, and non-English publications.

Data Extraction: Data on the antibacterial, anti-inflammatory, and antioxidant properties of *Punica granatum*, as well as its efficacy in treating gingivitis and periodontitis, were extracted.

**Figure 1:**
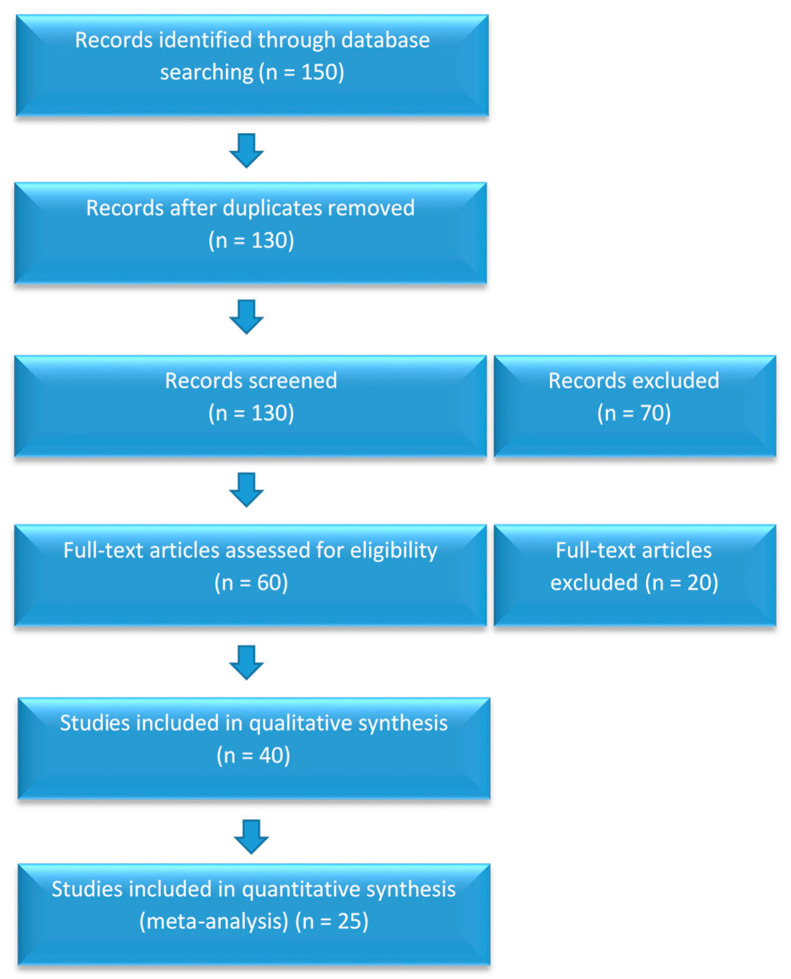
PRISMA flowchart of the study selection process.

Review Process: PRISMA guidelines were followed, and the search process is outlined in Figure 1.

### 2.1. Materials

Source of Nutraceuticals: Various parts of the pomegranate plant, including seeds, peels, and arils, contain significant bioactive components. The peel comprises 50% of the fruit’s weight and is rich in flavonoids, ellagitannins, proanthocyanidins, and minerals like potassium and iron [21]. The seeds are composed of water, sugars, vitamins (C, A, B), organic acids, and lipids [18,22].

### 2.2. Methods of Preparation and Consumption of Punica granatum

Pomegranate Juice: Known for its high vitamin C content, pomegranate juice contains phenolic compounds [23], such as punicalagin [24,25], and significant amounts of potassium, calcium [26], and antioxidants [27]. The juice is primarily composed of sugars, organic acids, polyphenols, and tannins [28,29], which contribute to its strong antioxidant properties [30,31].

Pomegranate Peels: Representing 43–60% of the fruit [32,33], the peels are rich in bioactive compounds like phenolics, flavonoids, and tannins. Traditionally used to treat ulcers and diarrhea [34], pomegranate peels have also shown anti-cancer [35,36,37] and anti-inflammatory effects [24,33,36,38,39,40].

Pomegranate Seeds: The seeds are known for their antimicrobial, anti-cancer, and antioxidant properties [35,41,42], containing anthocyanins, tannins [32,34,38,39,43], fatty acids, and phytochemical compounds like lignans and sterols [21,44,45,46].

Pomegranate Extract: The extract is obtained by processing the peels and seeds, yielding standardized components such as punicalagin, ellagic acid, and polyphenols [47,48]. These extracts are recognized for their high antioxidant capacity, exceeding that of green tea and red wine [49].

## 3. Functional Components of *Punica granatum*

**Figure 2:**
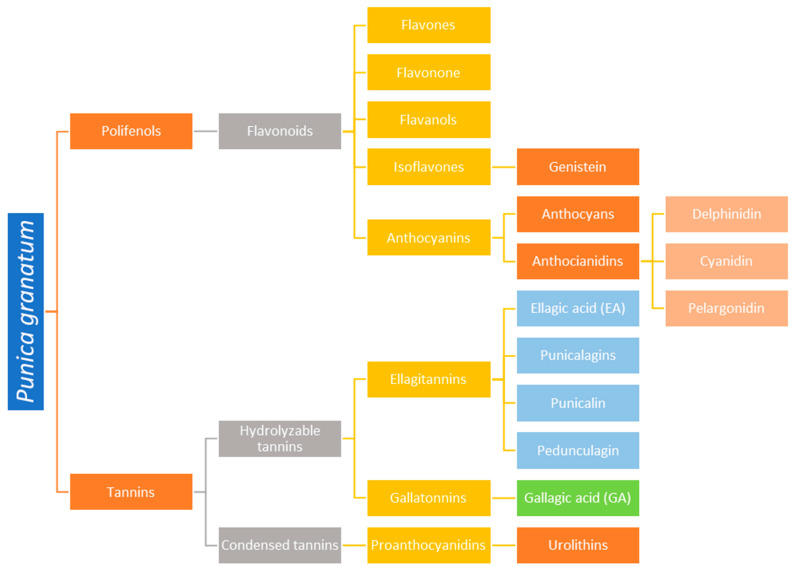
The content of polyphenols and tannins of *Punica granatum*.

Each part of the pomegranate plant, including the bark, seeds, flowers, and peels, offers unique health benefits, firmly establishing it as a significant nutraceutical food. The most prominent product derived from pomegranate is the juice, which is rich in polyphenols, vitamins, and minerals, contributing to its high antioxidant capacity. Pomegranate peel extracts have traditionally been used to treat ulcers and diarrhea, and recent studies highlight their anti-cancer and anti-inflammatory properties. The content of polyphenols and tannins of *Punica granatum* is presented in Figure 2.

### 3.1. Polyphenols

*Punica granatum* has a high polyphenol content that contributes to its antioxidant activity, as demonstrated in vitro. However, the bioactive substances responsible for antioxidant activity in vivo may differ from those present in the whole fruit, as these compounds are metabolized during digestion, resulting in the production of ellagic acid and urolithins. The health benefits of pomegranate are also attributed to anthocyanins and the unique fatty acid profile of pomegranate seed oil [50].

### 3.2. Flavonoids

Pomegranate is particularly rich in flavonoids, which are the primary polyphenols in the fruit [51,52,53]. These compounds have anti-inflammatory properties that promote good oral health, particularly by helping to prevent gingivitis [54]. The pericarp (peel) contains flavones, flavanones, flavanols, and tannins (such as punicalagin) that exhibit anti-inflammatory, anti-mutagenic, and antifungal activity [31]. Punicalagin, in particular, is an antioxidant that is three times more potent than those found in red wine or green tea [55].

### 3.3. Anthocyanins and Anthocyanidins

Anthocyanins are a class of flavonoids that serve as the main pigments in the plant kingdom. Their colors range from pink to red, purple, and blue, depending on the pH. Beyond their use as natural food colorants, anthocyanins have been widely studied for their potential health benefits, particularly in the prevention of cardiovascular, neurological, and other chronic diseases due to their antioxidant and anti-inflammatory activities [56,57,58].

Anthocyanidins in red fruits contribute to antioxidant activity, giving pomegranate juice superior bioactivity compared to purified polyphenols [19]. Pigmented fruits and vegetables, such as pomegranates, dark grapes, berries, and eggplants, are rich in delphinidin, a powerful antioxidant [59]. Bertuglia et al. described the mechanism by which delphinidin prevents endothelial dysfunction induced by oxidative stress in vivo [59]. Additionally, cyanidin and its glycosides, another type of anthocyanin, are commonly consumed through red wine, fruits, and vegetables, indicating a substantial daily intake of these compounds [60]. Another flavonoid, pelargonidin, is found in large quantities in raspberries, currants, and blueberries [61].

### 3.4. Fatty Acids

**Figure 3:**
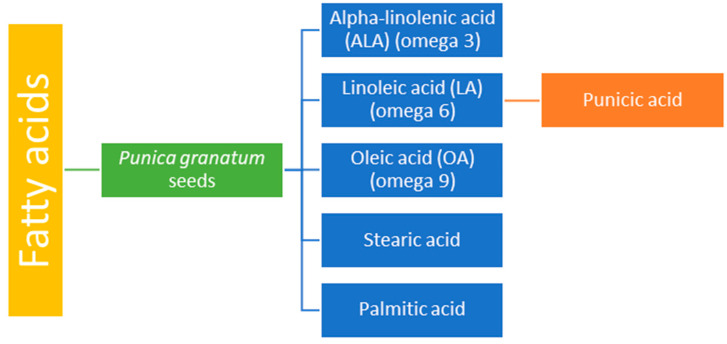
Fatty acid content of *Punica granatum* seeds.

Pomegranate seeds contain a variety of fatty acids, comprising 12–20% of their total dry weight. Among these, alpha-linolenic acid (omega-3), linoleic acid (omega-6), and oleic acid (omega-9) are present in significant amounts, along with stearic acid, which can help lower cholesterol levels, and palmitic acid [18] (Figure 3). The seeds are also rich in proteins, crude fibers, vitamins, minerals, pectins, sugars, polyphenols, isoflavones (such as genistein), coumestrol, and steroids (such as estrone) [18,22]. Due to these bioactive components, pomegranate seeds are used in the treatment of heart diseases, diabetes, obesity, cognitive imbalance, and cancer, as well as to improve male fertility [62,63,64].

Pomegranate seed oil is primarily composed of punicic acid and sterols, which possess nephroprotective properties [31,65]. Punicic acid, the main fatty acid in pomegranate, is a type of conjugated linoleic acid that has biological effects such as supporting weight loss and controlling diabetes. It may also help reduce the risk of breast cancer and prostate cancer [55].

### 3.5. Tannins

The tannins present in pomegranate are classified into two types: hydrolyzable tannins and condensed tannins [51,52,53]. Hydrolyzable tannins can be further divided into gallotannins, which hydrolyze to produce sugar and gallic acid, and ellagitannins, which hydrolyze to produce ellagic acid in addition to sugar and gallic acid [66,67]. Hydrolyzable tannins such as punicalagin, pedunculagin, and punicalin, along with glucose esters of gallic acid and ellagic acid, contribute significantly to the antioxidant activity of the whole fruit [29,51].

The primary hydrolyzable tannin present in pomegranate is punicalagin, while other hydrolyzable tannins include ellagitannins and gallotannins [68,69,70,71]. Research has shown that hydrolyzable tannins and polyphenols in pomegranate extract, particularly punicalagin and gallic acid, are likely responsible for the fruit’s antibacterial activity [72]. The antimicrobial activity of tannins is believed to be linked to their molecular structure and toxicity to bacteria. Tannins can damage bacterial cell walls and membranes [73,74].

In pomegranate leaves, major constituents include tannins such as punicalin and punicafolin, as well as glycoside flavones like luteolin and apigenin [31]. The leaves are known for their excellent antioxidant properties [22].

### 3.6. Triterpenoids

**Figure 4:**
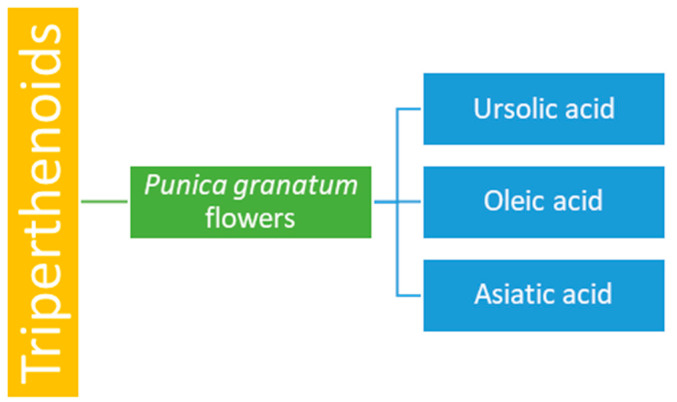
Triterpenoid content of *Punica granatum* flowers.

Pomegranate flowers contain triterpenoids such as ursolic acid, oleic acid, and asiatic acid (Figure 4), which possess antioxidant and hepatoprotective properties. These compounds are also used as a remedy for diabetes mellitus [31,75].

Ellagitannins and piperidine alkaloids are present in the roots and bark of pomegranate shrubs. The bark exhibits molluscicidal properties [31], and both the bark and roots are known for their anthelmintic and vermifuge properties [76]. The most beneficial components of pomegranate include ellagitannins, punicic acid, flavonoids, anthocyanidins, anthocyanins, and estrogenic flavones [31,77].

## 4. Major Active Substances of *Punica granatum*

**Figure 5:**
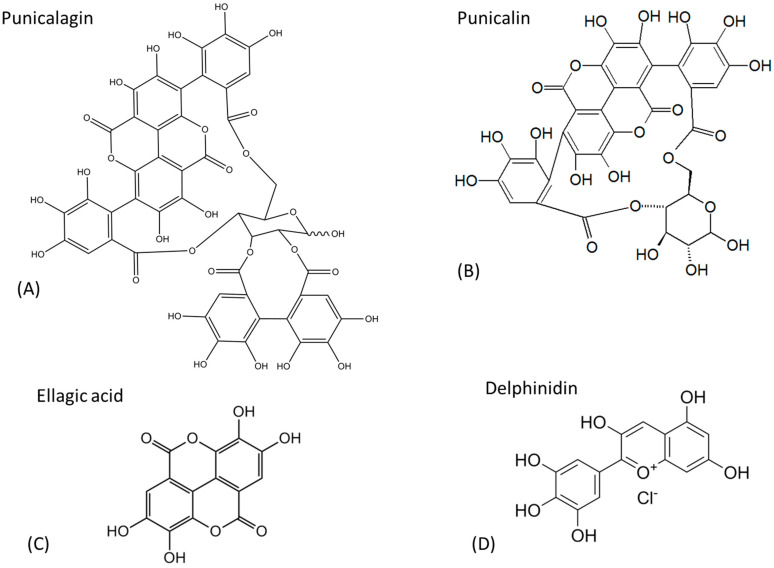
Chemical structures of the major active substances in *Punica granatum*.

The bioactive compounds responsible for *Punica granatum*’s health benefits include punicalagin, punicalin, ellagic acid (EA), and delphinidin, presented in Figure 5. These substances provide strong antioxidant, anti-inflammatory, and antimicrobial properties, making them crucial to the plant’s therapeutic potential. Punicalagin and punicalin are powerful polyphenolic compounds known for their strong antioxidant and anti-inflammatory properties. Ellagic acid is another potent antioxidant, recognized for its role in protecting cells from oxidative stress and reducing the risk of certain chronic diseases. Delphinidin, a type of anthocyanin, is responsible for the vibrant color of pomegranate and possesses antioxidant and anti-inflammatory effects, further enhancing the fruit’s therapeutic potential [78,79,80,81].

## 5. Therapeutic Effects of *Punica granatum*

### 5.1. Punica granatum in Diseases of the Oral Cavity

#### 5.1.1. Antibacterial Effects of *Punica granatum*

In dentistry, the antibacterial effects of pomegranate against *Streptococcus mutans* and *Streptococcus sanguinis* have been studied in vitro [74,82]. El-Sharkawy et al. (2019) evaluated the antibacterial activity of pomegranate extract against *Streptococcus mutans* and demonstrated that mouthwash containing pomegranate peel and juice was an effective antimicrobial agent. It significantly reduced the total number of bacteria in the saliva of children included in the study, comparable to the effect of a standard antiseptic (chlorhexidine 0.2%) [83].

DiSilvestro et al. (2009) showed that a pomegranate-based mouthwash reduced the number of microorganisms in bacterial dental plaque by approximately 84% [84]. In another study, the effectiveness of a pomegranate mouthwash was compared to a 0.2% chlorhexidine mouthwash in diabetic patients with gingivitis. After 21 days of use, both groups showed significant reductions in plaque and gingivitis indices, as well as probing bleeding [17]. According to Jacob et al. (2021), pomegranate extract mouthwash was less effective against *Streptococcus mutans* compared to chlorhexidine. However, it proved to be a natural and ecological alternative, effectively disrupting the activity of all tested microorganisms in their triple-blind randomized clinical trial focused on *Lactobacilli* and *Veillonella* [85].

Other studies, such as those by Kote et al. (2011), have demonstrated the effectiveness of pomegranate-containing mouthwashes against plaque microorganisms by significantly reducing the colonies of *Lactobacilli* (46%) and *streptococci* (23%) [19]. During a 24-h incubation period, *Punica granatum* gel, equivalent to 0.234% punicalagin, inhibited *S. mutans* and *S. sanguinis* but not *L. casei*, suggesting its potential use in the prevention of dental caries, as indicated by the other results [86].

Pomegranate juice has also been shown to be effective against bacterial dental plaque microorganisms, reducing colony-forming units (CFU). This was demonstrated by a significant reduction in the level of dental plaque microorganisms after rinsing with pomegranate juice [19]. The hydroalcoholic extract of the fruit similarly acts on bacterial dental plaque microorganisms, reducing the microbial load [87]. Another study examined the effects of rinsing the mouth with PomElla^®^ pomegranate extract in young adults, finding that the treatment favorably modulated salivary values, thus supporting oral health and preventing gingivitis [85].

Ghazi et al. (2020) evaluated the effect of pomegranate extract in mouthwash as a treatment for gingivitis in patients with type 2 diabetes. Both biochemical parameters (IL-1β, salivary AST) and clinical parameters (PI, GI, BOP) in the three groups included in the study were significantly reduced after 14 days of treatment. However, salivary IL-1β in the group that used chlorhexidine mouthwash (0.12%) did not show a significant reduction compared to the group that used pomegranate peel extract (6.25%) and the group with pomegranate peels and arils extract (12.5%) [21].

However, the antibacterial effects of pomegranate against periodontogenic microbial species have not been conclusively demonstrated, according to Laleman et al. (2020) [88]. In a clinical trial by Kiany et al. (2016), pomegranate mouthwash was found to be as effective against plaque as Persica and Matrica mouthwashes, two herbal mouthwashes commonly used in Iran, made from *Salvadora persica* extract and *Matricaria chamomilla*, respectively [89].

#### 5.1.2. *Punica granatum* and Gingivitis

Several clinical trials suggest that pomegranate extract significantly reduces plaque and gingival inflammation. *Punica granatum* extract has shown significant improvements in patients with gingivitis, reducing plaque index and bleeding on probing. Clinical trials have shown comparable effectiveness to standard antiseptic mouthwashes. Pomegranate mouthwash improved gingival health, comparable to conventional mouthwashes like chlorhexidine. The results obtained by Pasupuleti et al. (2023) suggest that pomegranate fruit and pomegranate peel extract may serve as promising alternatives in the management of chronic gingivitis and oral ulcers as adjuncts to scaling and root planing (SRP) procedures. They have the potential to be competitive alternatives to modern pharmaceutical products [90]. Eltay et al. (2021) demonstrated the adjuvant effect of a pulsatile oral spray containing 5% pomegranate extract. The irrigation solution effectively reduced plaque index (PI), gingival index (GI), and interleukin-1β (IL-1β) values in patients with chronic gingivitis [14]. Pomegranate mouthwash has been shown to improve gingival health by reducing the PI (Tureskey–Gilmore–Glickman modification of the Quigley–Hein Index) and bleeding on probing (BOP) indices, with effects comparable to those of two commonly used herbal mouthwashes [89].

#### 5.1.3. *Punica granatum* and Periodontitis

Pomegranate extracts used as adjuncts to scaling and root planing (SRP) were shown to reduce inflammatory markers and improve periodontal health. In patients with periodontitis, pomegranate extract combined with conventional scaling and root planing has demonstrated reductions in inflammatory markers and clinical symptoms. Sastravaha et al. (2003) demonstrated that clinical symptoms of chronic periodontitis were reduced in 20 patients following scaling and root planing, combined with the subgingival delivery of biodegradable chips containing *Centella asiatica* and *Punica granatum* [91]. Another study comparing adjunctive topical treatment with extracts of *Centella asiatica* and *Punica granatum* to standard supportive periodontal therapy revealed significant improvements in interleukin-1 levels and clinical parameters of chronic periodontitis in 15 patients [92]. In 2005, Sastravaha et al. presented the efficacy of a toothpaste containing pomegranate extracts as a complementary treatment to routine periodontal therapies, demonstrating in vitro that pomegranate flavonoids have antibacterial properties against microorganisms responsible for gingivitis [92].

#### 5.1.4. *Punica granatum* and Stomatitis

Pomegranate-based gels have shown accelerated healing in patients with aphthous stomatitis. In one study, pomegranate gel significantly reduced pain and healing time compared to placebo. Tavangar et al. (2019) clinically evaluated the effects of a mucoadhesive pomegranate gel administered to 60 patients with moderate aphthous stomatitis. They showed that, compared to the groups receiving Triadent product and placebo gel three times a day, local pain in stomatitis subsided more rapidly, and healing was accelerated [93]. Ghalayani et al. (2013) obtained similar results by administering a 10% pomegranate gel to patients with recurrent aphthous stomatitis (RAS), significantly reducing pain relief time, time to complete healing, and the “visual analog scale” score [94].

Pomegranate peel gel, used in another clinical trial, had comparable effects in patients with RAS, reducing pain, ulcer size, and healing time [95]. Alcoholic and aqueous extracts of *Punica granatum var. pleniflora* also reduced the total duration of complete treatment [96]. An in vitro study conducted by Abdollahzadeh et al. (2011) showed that methanolic pomegranate extract (MEPGP) could be used to control common oral pathogens responsible for caries, stomatitis, and periodontal diseases. However, further phytochemical studies are needed to identify the specific antibacterial compounds in pomegranate involved in these effects [10].

Several authors have investigated the effects of pomegranate gel in prosthetic stomatitis [74,75,97]. César et al. (2003) observed a reduction in prosthetic stomatitis caused by candidiasis in 76.7% of patients treated with *Punica granatum* Linne gel three times a day for 15 days, highlighting its antifungal effects [75]. Periodontal gel with pomegranate peel extract appears to be as effective as miconazole gel, a topical antifungal agent [97].

Vanconcelos et al. (2003) conducted an in vivo study using pomegranate gel as an antifungal agent against candidiasis associated with dental stomatitis and found that symptoms were resolved and overall oral health was improved [75]. In 2006, Vanconcelos et al. investigated the antimicrobial effect of pomegranate gel against *Streptococcus mutans*, *Streptococcus mitis*, and *Candida albicans* and found that pomegranate gel effectively inhibited microbial adhesion [74]. The findings suggest that pomegranate gel could be used to control the adhesion of various microorganisms in the oral cavity responsible for dental caries, stomatitis, and periodontal disease.

The pomegranate fruit, referred to by Jurenka et al. (2008) as “a pharmacy in itself”, exhibits numerous medicinal properties, including antifungal, antiviral, immunomodulatory, bactericidal, astringent, diuretic, and vermifuge effects [16]. Numerous studies have demonstrated that pomegranate extracts or the fruit itself can effectively manage inflammation [14]. *Punica granatum* has been widely used in the treatment of cardiovascular diseases (hypertension, atherosclerosis), metabolic disorders (diabetes, obesity, hyperlipidemia), respiratory conditions (asthma, bronchitis, cough), and dental issues (coagulation disorders, dental stomatitis) [10,16,78,98,99].

The recent development of phytotherapy in dentistry has made it possible to use the medicinal properties of plants and herbs to treat a variety of oral conditions. This alternative, natural, safe, and affordable therapy offers an effective substitute for synthetic drugs [100].

#### 5.1.5. *Punica granatum* Mouthwash

Studies demonstrate that *Punica granatum* mouthwashes are highly effective in reducing bacterial load and plaque formation. For instance, pomegranate-based mouthwashes reduce bacterial plaque by 84%, similar to the effects of chlorhexidine, a standard antiseptic.

The long-term use of chemical and pharmaceutical preparations is known to pose health risks. Therefore, it is sensible to seek more plant-based alternatives. Pomegranate extract mouthwash is gaining popularity as a viable option, especially in rural areas and less developed countries, where access to conventional oral care products may be limited [101,102].

- *Punica granatum* gels
- *Punica granatum* toothpaste
- Other pharmaceutical forms of *Punica granatum*

Recently, Gawor et al. (2023) evaluated the effectiveness of a pomegranate additive in water to limit the accumulation of bacterial plaque and tartar in dogs. Their results showed, for the first time, that this additive reduces bacterial deposits and improves gingival health, recommending the tested product for home use due to its time-saving and convenient properties [103]. A previous study by Silva et al. (2020), also conducted in dogs, formulated and evaluated a mucoadhesive oral ointment (orabase) containing pomegranate peel extract as an adjuvant for oral hygiene. The results suggest that the product derived from pomegranate peel extract is a viable option for improving oral hygiene, helping to reduce the bacterial component of dental plaque in experimental animals [104]. An overview of the studies reviewed is presented in Table 2.

#### 5.1.6. Other Health Benefits of *Punica granatum* in Comorbidities

Beyond oral health, pomegranate has demonstrated potential benefits for cardiovascular health, diabetes management, and cancer prevention. Traditional uses of pomegranate bark in treating diarrhea, inflammation, and parasitic diseases are well documented. Pomegranate has been formulated into various products such as mouthwashes, gels, and toothpastes, which show effectiveness in maintaining oral hygiene and reducing microbial loads.

### 5.2. Antioxidant Effects of Punica granatum

Pomegranate contains powerful antioxidants, including polyphenols, hydrolyzable tannins, and anthocyanins, which neutralize free radicals and protect against oxidative stress, reducing the risk of chronic diseases like cancer and cardiovascular conditions [49]. Studies show that pomegranate juice has a higher antioxidant capacity than green tea or red wine, largely due to its rich content of flavonoids and anthocyanidins such as delphinidin, cyanidin, and pelargonidin [16,105]. The antioxidant activity of pomegranate extends to scavenging hydroxyl radicals and superoxide anions [15,16], and methanolic extracts from the peel have demonstrated broad antioxidant potential through various assays [19,106]. Regular consumption of pomegranate juice has been linked to reduced oxidative stress, lowered systolic blood pressure [107], and enhanced overall heart health [108]. Additionally, the antioxidant properties are stronger when the juice is extracted from the whole fruit, as the peel contributes to antibacterial and astringent effects beneficial for oral health [109].

#### 5.2.1. Antioxidant and Anti-Inflammatory Effects of *Punica granatum*

The high polyphenol and flavonoid content of pomegranate contributes to its potent antioxidant activity, which has been shown to neutralize harmful free radicals, reduce inflammation, and enhance oral tissue health. From the perspective of pomegranate, the exploration of its bioactive compounds through neural networks in otolaryngology opens up new possibilities for enhancing treatment outcomes, but challenges such as data standardization, extraction of relevant biomarkers, and integration of traditional knowledge with advanced machine learning techniques remain significant hurdles [110].

#### 5.2.2. Topical Anti-Inflammatory and Analgesic Effects of *Punica granatum*

In addition to its antibacterial and antioxidant properties, pomegranate extract has demonstrated topical anti-inflammatory and analgesic effects. It works by preventing leukocyte infiltration and modulating the levels of pro-inflammatory cytokines such as interleukin-1β (IL-1β) and tumor necrosis factor-alpha (TNF-α), according to research by Mo et al. (2013) [111].

#### 5.2.3. Anti-Hemorrhagic and Astringent Effects of *Punica granatum*

Kiany et al. (2016) also highlighted the astringent and anti-hemorrhagic properties of *Punica granatum* when used as a mouthwash. The reduction in gum bleeding was comparable to that achieved by other mouthwashes containing standard plant extracts [102].

## 6. Discussion

In this review, we used PubMed and Google Scholar for the literature search. PubMed was selected for its comprehensive coverage of peer-reviewed biomedical studies, ensuring high-quality sources relevant to health and oral diseases. Google Scholar was included for its broader range, capturing gray literature and studies not indexed in PubMed.

While additional databases like Scopus or Web of Science could have been used, we focused on PubMed and Google Scholar to balance comprehensiveness and relevance. We acknowledge this may limit the scope, but these databases provide a strong foundation for the study’s objectives.

The review was conducted by analyzing the specialized literature on the bioactive properties of pomegranate-derived products and their effects on conditions of the oral cavity. To identify the most relevant articles, the search strategy employed a combination of keywords, including “*Punica granatum*” or “pomegranate”, and “oral diseases”, “gingivitis”, “periodontitis”, “stomatitis”, and “nutraceutical”. No time constraints were applied to the database search.

Compared to other natural extracts [112,113,114], this review investigates the antibacterial, antioxidant, and revitaminizing effects of pomegranate. Periodontal diseases are correlated with numerous chronic diseases [115,116,117] and can significantly affect the quality of life.

The findings from this review underscore the potential of *Punica granatum* as a viable natural alternative for treating oral diseases such as gingivitis and periodontitis. Pomegranate’s antibacterial, anti-inflammatory, and antioxidant properties offer substantial benefits for oral health, making it a promising candidate for use in dental products like mouthwashes and gels. However, the limitations of current research include a lack of standardized dosages and long-term safety data. More clinical trials are needed to determine the optimal formulations and assess potential adverse effects.

Strengths and Limitations: Despite the thorough approach, the review is constrained by its reliance on only two databases, which may have excluded relevant studies from other sources or unpublished data, potentially limiting the breadth of the findings.

This review has strengths in its comprehensive coverage of both clinical and experimental studies, but it is limited by its reliance on two databases and the exclusion of non-English studies, which may have led to missed relevant research. Future reviews should aim for a broader search strategy.

## 7. Conclusions

Extracts from different components of the medicinal plant *Punica granatum* have antibacterial, anti-inflammatory, antioxidant, and astringent properties, offering favorable potential in limiting the pathogenic oral microbiome and preventing or treating oral cavity conditions such as gingivitis, periodontitis, and stomatitis. These bioactive compounds, derived from pomegranate seeds, peels, and juice, have shown efficacy in reducing plaque, inhibiting the growth of harmful microorganisms, and promoting overall oral health. Further clinical trials and in vivo studies are needed to fully understand the optimal formulations, dosages, and long-term effects of *Punica granatum* in dental care. The integration of pomegranate-based products into oral hygiene practices could provide a natural, effective, and accessible alternative to synthetic pharmaceuticals, particularly in regions with limited access to conventional dental care.

Pomegranate extracts exhibit antibacterial, anti-inflammatory, antioxidant, and astringent properties, demonstrating potential in treating oral diseases such as gingivitis, periodontitis, and stomatitis. These natural bioactive compounds can effectively reduce plaque and harmful microorganisms, offering a promising alternative to synthetic pharmaceuticals. Further clinical trials are required to confirm the optimal formulations and long-term safety of pomegranate-based dental products.

## Figures and Tables

**Table 1:** Inclusion and exclusion criteria for study selection.

Inclusion Criteria:	Exclusion Criteria
Peer-reviewed studies	Non-peer-reviewed articles
Focus on *Punica granatum* and oral diseases	Studies not focused on oral health
English language	Articles in languages other than English
In vitro, in vivo, and clinical trials	

**Table 2:** Overview of studies investigating the effects of *Punica granatum* in various pharmaceutical forms for oral health.

Author and Reference	Type of Study	Form of Preparation	Comparison Substance	Main Results
Kiany et al. (2016) [89]	Double-blind clinical trial Number of participants: 104 total 46 males 58 females Disease: mild to moderate gingivitis Before treatment: scaling and root planning (SRP) Treatment: 2 weeks after SRP Time of treatment administration: 1 month	Mouthwash with extract of seeds of fresh pomegranate fruits Method of preparation: Cold pressing of fresh pomegranate fruits without the peel, seeds separated from extract Distilled water Preservative: 0,4% methylparaben 0,04% sodium saccharine Pasteurized Dosage: drop Container: 60 mL	Persica mouthwash with herbal extract of *Salvadora persica* Matrica mouthwash with herbal extract of *Matricaria chamomilla* Placebo	↓ Plaque index (PI): Tureskey–Gilmore–Glickman modification of Quigley–Hein ↓ Bleeding index (BOP): Lenox
Menezes et al. (2006) [87]	Clinical and in vitro Number of participants: 60 healthy patients using fixed orthodontic appliances 27 males 33 females Disease: no disease dental plaque collection Before treatment: no tooth brushing for 24 h before dental plaque collections Treatment: Time of treatment administration: 1 min mouthrinse with 15 mL of either HAE, CHX, or distilled water	Hydroalcoholic extract (HAE) from *Punica granatum* fruits on dental plaque microorganisms Dental plaque samples were diluted in phosphate-buffered saline (PBS) plated on Mueller-Hinton agar and incubated for 48 h at 37 °C	Standard CHX mouthwash Distilled water	HAE was very effective against dental plaque microorganisms by ↓ colony-forming units per milliliter (CFU/mL) ↓ CFU/mL by 84% for HAE before mouthrinse 154.0 +/− 41.18 after mouthrinse 25.4 +/− 7.76 ↓ CFU/mL by 79% for CHX before mouthrinse 208.7 +/− 58.81 after mouthrinse 44.0 +/− 15.85 ↓ CFU/mL by 11% for distilled water before mouthrinse 81.1 +/− 10.12 after mouthrinse 71.9 +/− 8.68 HAE also presented antibacterial activity against selected microorganisms
Millo et al. (2017) [86]	In vitro Type of bacteria: *Streptococcus mutans* *Streptococcus sanguinis* *Lactobacillus casei*	*Punica granatum* gel Method of preparation: High-performance liquid chromatography (HPLC) was used for the identification and quantification of the chemical marker punicalagin Minimum bactericidal concentration (MBC) and time-kill assay (TKA) were investigated tested by measuring the zones of inhibition through the agar well diffusion method	2% CHX gel blank gel	PG gel equivalent to 0.234% punicalagin inhibited *S. mutans* and *S. sanguinis* within the 24 h incubation period but did not inhibit *L. casei* within the 24 h incubation period has the potential to be used for caries prevention MBC for *S. mutans* 250 mg/mL MBC for *S. Sanguinis* 125 mg/mL MBC for *L. casei* 500 mg/mL The TKA of 500 mg/mL aqueous PG extract showed total inhibition of *S. mutans*, *S. Sanguinis*, and *L. casei* at 6, 1, and 24 h contact time

↓ = reduction

## Data Availability

Data sharing is not applicable to this article as no datasets were generated or analyzed during the current study.
